# Experiences from leading parental education groups: Perceived difficulties and rewards as an indication of skill acquisition

**DOI:** 10.1177/13674935211000940

**Published:** 2021-03-12

**Authors:** Michael Rosander, Karin Forslund Frykedal, Mia Barimani, Anita Berlin

**Affiliations:** 1 4566 Linköping University, Linkoping, Sweden; 2 97092 Karolinska Institutet, Stockholm, Sweden

**Keywords:** Parental education groups, skill acquisition, learning, leadership, prenatal education, postnatal education

## Abstract

Developing skills in a professional setting is linked to practical experience. The relationship between experience and acquisition of skills can be seen as a transition from novice to expert. In a nursing setting, this has been studied using the Dreyfus model of skill acquisition. The aim was to investigate how experience influences midwives’ and child healthcare nurses’ views of difficulties and rewards in working with parental education groups. The study has a cross-sectional design with a mixed methods approach. A total of 437 midwives and child healthcare nurses answered a web-based survey. First, a qualitative analysis was carried out, and then patterns of experience were analysed. The results showed that less experience as a leader corresponds to a greater focus on one’s own role and on personal benefits from working with parents, but not on the specific context of the group. With experience, leaders had a greater focus on the group itself and rewards of making it function well. Not being able to take the current group and the specific context into account when working as a leader reduces possibilities of achieving a well-functioning group and the goals of the parental education.

## Introduction

Getting support in the transition to parenthood is important for expectant and new parents (Barimani et al., 2017). Group-based programmes focussing on relations, allowing parents to be active participants and giving opportunities to build social networks are most likely to provide effective support (McMillan et al., 2009). Lefevre et al. (2016) showed that group leadership is an important factor in how effective the support is. However, midwives and child healthcare nurses are often inadequately prepared and supported in taking on a leader’s role for this type of group (Barlow et al., 2009; Lefevre et al., 2015; Sarkadi, 2009). The current study focusses on experienced difficulties in this role, as well as what is perceived as rewarding from a leader’s perspective.

Group-based programmes for parenting support are offered by different disciplines aimed at all ages of a child (Olofsson et al., 2016). In this study, we focussed only on the support given during pregnancy and the first months up until a year after childbirth to first-time parents. From an international perspective, in this time span, parental education groups typically take place before and directly after birth (perinatal) and are led by midwives (Gagnon and Sandall, 2007). In Sweden, where the current study was carried out, most expectant and new parents who attend primary healthcare are offered parental education in groups. These groups continue after the perinatal period. In most cases, nurses in child healthcare create new groups of parents with newly born children and take over as providers of parental education from midwives in antenatal care (Barimani et al., 2017; Fabian et al., 2006). Sometimes, a group stays together throughout pregnancy and early parenthood (up to about one year after childbirth). In a Swedish context, these groups are most often referred to as ‘parental groups’.

Parental education in Sweden has a focus on enhancing competence and coping capabilities of parents and helping create networks between parents (Berglund et al., 2016; National Handbook of Child Health Services, 2020). The parental education offered is in principle the same programme based on these common national guidelines. In this study, there is a focus on the leader role that midwives or nurses in child healthcare take on in parental education groups and not on their profession as a nurse or a midwife; hereafter, we will refer to both as leaders of parental education groups.

Being a leader of a parental group often requires knowledge and skills beyond that which is directly linked to being a midwife or a nurse in child healthcare. Nurses and midwives have knowledge and skills in their specific professional areas, but not necessarily of group dynamics. Nor is it obvious that they would have teaching skills or an ability to lead educational groups (Forslund Frykedal et al., 2019; Lefevre et al., 2016). There are also discrepancies regarding what they say they do and what they actually do when leading parental education groups, describing a more parent-centred facilitator role while taking on more of an expert role in reality (Berlin et al., 2018). Research on skills needed in this setting is sparse. Nursing leadership typically involves a management role of resources or staff (Cummings et al., 2010; Curtis et al., 2011; Pearson et al., 2007) and not leadership skills needed for group work in an educational setting. Group work skills in training often involves working as part of a group or team (Chakraborti et al., 2008), not necessarily being in a leader role.

Leadership in parental education groups has been associated with a lack of confidence in its delivery (Barlow et al., 2009; Forslund Frykedal et al., 2019; Forslund Frykedal et al., 2016; Lefevre et al., 2015). There is also a lack of specialised training in teaching skills, as well as leadership skills specifically aimed at group work (Ahlden et al., 2008; Barlow et al., 2009). Wiener and Rogers (2008) suggested that group facilitation training should be an integral part of basic training for midwives. Group supervision can help develop and increase midwives’ professional competence when working with pregnant women and their need for emotional support (Severinsson et al., 2010). This increased competence included an increase in awareness and sensitivity in the professional role, aspects which are also highly relevant to development of the leader role in parental groups.

## Background

Developing skills in a professional setting is linked to practical experience in the workplace (Dall’Alba and Sandberg, 2006). The relationship between experience and acquisition of skills can be seen as a transition from novice to expert. In a nursing setting, this has been studied by Benner (1982), (2004) based on a model developed by Dreyfus and Dreyfus (1980): the Dreyfus model of skill acquisition. The Dreyfus model has been applied to various aspects of nursing and nursing education to understand the relationship between skill acquisition on the one hand and teaching effort and framework for planning or training on the other (Blum, 2010; Green et al., 2009). The model articulates, for example, progress of clinical knowledge and skills (Atsalos et al., 2014; Benner, 2004; Benner et al., 2009; Carraccio et al., 2008; Khan and Ramachandran, 2012), decision-making (Blum, 2010), problem-solving (Pena, 2010) and the nurse educator role (Ramsburg and Childress, 2012).

### The Dreyfus model of skill acquisition

To acquire a new skill requires, according to Dreyfus and Dreyfus (1986, 1980), either imitation with trial and error or seeking aid of an instructor or instructional manual. Dreyfus and Dreyfus (2009) later argued that practice without theory, and vice versa, alone cannot produce fully skilled behaviour. The Dreyfus model includes five stages of skill acquisition: novice, advanced beginner, competent, proficient and expert. The model emphasises primarily intuition and reflection as critical in the development of skills. Development through the five stages can be seen as a result of a successive transformation of four mental functions: recollection, recognition, decision and awareness (Dreyfus and Dreyfus, 1980). Each function has its own level at every stage. There is an assumption that learning occurs in steps or stages. The skills of one stage must be acquired and incorporated before moving forward to the next stage in the learning process. The model focuses on situated use of skills in a delimited area (for example) within a profession (Benner, 2004). Experiences from this specific area are central to the understanding of skill acquisition. To better understand how skills are acquired, Dall’Alba and Sandberg (2006) suggested an expanded model in which, in addition to experience from relevant situations, they included an embodied understanding of the practice as a necessary aspect to understand skill acquisition.

The skills referred to in this study are pedagogical and group psychology skills used by midwives and nurses in parental education groups. Forslund Frykedal and Rosander (2015) described that pedagogical skills broadly comprise how leaders manage to teach their knowledge within their area of expertise and how they manage to adapt their teaching to match the needs of the group, and that group psychology skills broadly comprise how to manage, as a leader, to interact with a group and how to handle group dynamics.

### Aim

To investigate the role of experience in how midwives and child healthcare nurses look at difficulties and rewards of working with parental groups in terms of skill acquisition.

## Methods

### Design

This study had a cross-sectional design with a mixed methods approach (Tashakkori and Creswell, 2007) involving open-ended survey questions first analysed using conventional qualitative content analysis (Elo and Kyngas, 2008; Hsieh and Shannon, 2005). The resulting categories were then subjected to a quantitative analysis based on a summative content analysis (Elo and Kyngas, 2008) and interpreted using the Dreyfus model of skill acquisition (Dreyfus and Dreyfus, 1980, 1986; Dreyfus, 2004).

### Data collection and participants

A web survey was created with the survey tool Survey&Report (Artologik, 2015). An email with a link to the survey was sent to all midwives in antenatal care and nurses in child healthcare in Stockholm County. To get access, we first contacted the health and medical care administration in Stockholm County. They found the study important and referred us to each primary care unit in the county who provided email addresses of all active midwives and nurses in child healthcare at each unit.

The questionnaire contained a brief description of the purpose, and that it was anonymous, with participation being voluntary. There were three seven-point Likert scale items capturing to what degree respondents see themselves as leaders and teachers for parental groups, as well as how confident they were in their role. The questionnaire also included two questions with open answers: ‘What is the biggest challenge or difficulty for you when working with parental education groups?’ and ‘What is most rewarding when working with parental education groups?’. There were also questions about age, number of years they had worked with parental groups, how many groups they start in a year, how many times they usually meet each group and if they worked in antenatal care or in child healthcare.

### An estimate of experience

To obtain an estimate of experience, a combined score of (a) how long they had been working with parental education groups, (b) how many, on average, they start each year and (c) how many times they usually meet each group was calculated. The mean number of years working with parental education groups were 10.9 years (SD = 9.0, maximum 43 years). The leaders started on average 3.6 groups each year (SD = 0.8, ranging from 1 to 4). On average, they met with a group 4.2 times (SD = 2.1, ranging from 1 to 10). Multiplying these three measures resulted in an estimate of experience in terms of how many times respondents have had a session with a parental education group throughout their entire professional career. The mean experience was 171.8 group sessions (SD = 186.7, maximum 960 group sessions).

### Analysis of data

The analysis was conducted in a number of steps. First, we conducted a qualitative content analysis (Elo and Kyngas, 2008; Hsieh and Shannon, 2005) based on the answers to the two open questions in the questionnaire (see Tables 1 and 2). Second, for each participant, and for each subcategory from the content analysis in step 1, we noted if the participant had expressed the subcategory or not. Third, for each subcategory, we compared the level of experience for the leaders who expressed a subcategory to those who did not (see Table 3). Below, the qualitative and quantitative analyses are described in more detail.

**Table 1.** Qualitative main and subcategories for the difficulties when working with parental groups.

Category	Description
*A. Managing group processes (main)*	
Manage distractions	To hold one’s own as a leader and the parents' focus when there were too many participants in the group and, for example, when parents walked around the room to comfort their crying babies.
Creating involvement	Meeting ‘silent’ groups: To capture the interest of the participants, where they all talk, exchange experiences, reflect and are dedicated and enthusiastic. To get the whole group acting as a unit where everyone feels they are seen and heard.
Creating balance	To handle parents' differences, for example, differences in age, culture, language and education, and to create balance in the group when someone was dominant. This also involves getting the father to feel part of the group.
Manage emotions	To manage the influence of anxious and sad parents on the rest of the group and to handle parents who were questioning the group and the leader and those who expressed what was perceived as extreme and inappropriate opinions.
Creating security	To create a safe atmosphere with an open and tolerant climate where parents felt safe, comfortable and able to ask questions.
*B. Uncomfortable in the role (main)*	
Finding the right level of content	To find the right level of the content on the basis of everyone’s different needs, backgrounds and knowledge. To find the right balance between the parents' needs and wishes and what needs to be conveyed.
Manage professional shortcomings	Having large and many new groups becomes monotonous and repetitive making it hard to be enthusiastic. Lack of teaching and academic skills, and of up to date materials making it difficult to respond to well-informed parents. To not have access to or not being able to handle the appropriate technologies, such as computers and PowerPoint.
Manage personal shortcomings	To stand in front of a group when one, as a leader, feels shy, has low self-esteem and has difficulties taking up and leading discussions on sensitive topics.
Finding the right level of mediation	To find good material and to convey it in a way that is easy to understand, interesting and fun. To keep focused although expected responses from the parents are absent (expecting a lecture).

**Table 2.** Qualitative main and subcategories for the rewarding aspects of working with parental groups.

Category	Description
*A. Well-functioning groups (main)*	
Exchange of experience	When groups can share experiences, reflect together and, in some ways, teach each other.
Active parents	When parents are active, ask many questions and take part in lively discussions. When parents show curiosity, commitment and interest.
Cohesion	When a good cohesion is created in the group where parents feel connected with each other, feel safe and think that the group is important; where parents dare to talk about difficult issues with each other.
Lasting friendship	When parents create their own networks and continue to socialise and support each other after the parent group is concluded.
*B. Personal benefit (main)*	
Getting to know the parents	To get to know the parents, hearing their thoughts and expectations.
Confirmation	Satisfied parents giving positive responses and they find the meetings useful.
Challenge oneself	To meet new and different groups of parents each time, also getting to meet people from different cultures.
Own development	Satisfaction of imparting knowledge, providing information, to explain and answer questions but also to provide inspiration and delight.

**Table 3.** Median and interquartile range for experience for those who are represented in a subcategory and those who are not (n), and comparison between the them using Mann–Whitney U tests with difference estimate and 95% confidence intervals for the median differences based on Hodges-Lehmann.

	Not in the category			In the category			Difference	95% CI	
	Md	IQR	*n*	Md	IQR	*n*	Estimate	Lower	Upper
**Difficulties**									
*A. Managing group processes*									
Managing distraction	100	200	420	90	299	12	0	−80	72
Creating involvement	84	229	188	102	196	244	−12	−32	4
Creating balance	100	200	387	126	228	45	−26	−60	4
Managing emotions	96	200	412	264	296	20	−128	−208	−52
Creating security	96	181	394	304	384	38	−182	−252	−108
*B. Uncomfortable in the role*									
Managing professional shortcomings	108	224	383	48	112	49	32	8	66
Finding the right level of content	111	248	344	60	122	88	32	10	58
Managing personal shortcomings	100	204	422	90	174	10	24	−40	100
Finding the right level of mediation	100	200	396	120	258	36	−18	−60	16
**Rewards**									
*A. Well-functioning groups*									
Active parents	100	200	320	110	254	112	−8	−28	12
Exchange of experience	96	224	355	112	168	77	−2	−28	20
Cohesion	82	182	332	168	244	100	−60	−92	−32
Lasting friendship	96	186	353	182	272	79	−40	−84	−12
*B. Personal benefit*									
Confirmation	104	237	386	56	104	46	36	8	72
Getting to know the parents	102	224	406	56	85	26	32	0	80
Own development	100	214	417	60	144	15	24	−12	72
Challenge oneself	100	212	411	80	136	21	18	−24	80

### Qualitative analysis

For this study, the answers to the open-ended questions on difficulties and rewards when working with parental education groups were used. The two questions were analysed separately using conventional qualitative content analysis (Elo and Kyngas, 2008; Hsieh and Shannon, 2005). All four researchers first discussed and agreed on a common analytic procedure to increase trustworthiness of the study. The analysis was then, first, carried out individually, then compared, refined and revised together in discussions to reach a consensus on data interpretation. The main literature review including the Dreyfus model was conducted after all categories and subcategories had been constructed, to guarantee their relevance. The aim of the study was not to categorise the midwives and nurses in this study using the Dreyfus model but to use the framework to understand the result.

### Quantitative analysis

**Figure 1.**
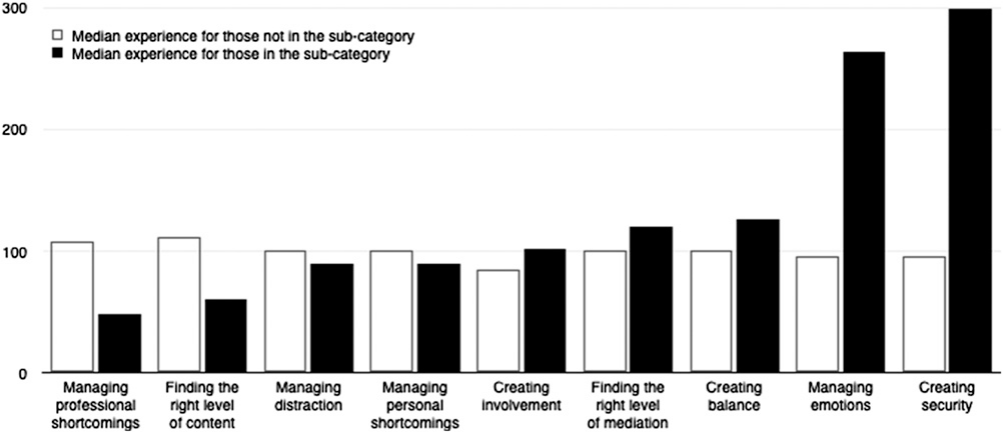
Levels of experience for the difficulties ordered by the median difference of experience between those expressing a category and those who do not.

**Figure 2.**
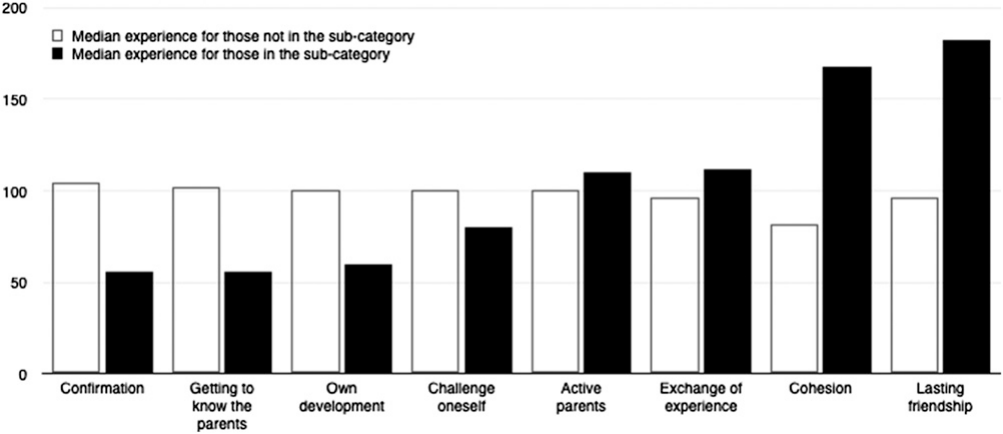
Levels of experience for the rewards ordered by the median difference of experience between those expressing a category and those who do not.

The qualitative result was divided into difficulties and rewards of working with parental education groups. Both difficulties and rewards have main categories and a number of subcategories. Each subcategory was analysed using a Mann–Whitney U test comparing the level of experience between leaders who expressed the difficulty or reward and those who did not. That is, we went back to the raw data and did a summative content analysis based on the categories found in the conventional content analysis (Elo and Kyngas, 2008; Hsieh and Shannon, 2005). Confidence intervals for median differences were calculated using Hodges–Lehmann estimates. Each set of subcategories (for difficulties and for rewards separately) could then be examined together for patterns of how difficulties and rewards of working with parental education groups varied in terms of experience. The order of subcategories for difficulties and for rewards was based on the empirical findings (median experience of the leaders expressing the difficulty or reward). The median experience used for the ordering can be found in Table 3 under the heading ‘In the category’ and is the order the subcategories are presented in Figures 1 and 2. This order was also used as a basis for a recoding of each individual’s answers into two new variables (difficulties rank 1–9 and rewards rank 1–8). As an example, if a leader had expressed the difficulty Managing distraction’ the rank for that leader would be 3 as leaders expressing ‘Managing distraction’ has the third lowest experience level (as presented in Table 3). In case an individual had expressed more than one subcategory, the highest rank was used. The new variables were correlated with experience using the Spearman rank correlation with 95% bootstrapped confidence intervals. For all quantitative analyses, we used IBM SPSS 26.

### Ethical considerations

All participants were informed about the study beforehand and answered anonymously via a web questionnaire. The questions covered only their professional role as leaders for parental education groups. The study was approved by the regional Research and Ethics Committee at Linköping University (# 2013/359-31).

## Results

A total of 437 responses were received, out of 834, giving a response rate of 53% (152 midwives and 285 nurses in child healthcare). The qualitative analysis resulted in three main categories of *difficulties* the leaders experienced when working with parental education groups: (a) managing group processes, (b) uncomfortable in the role as leader of the group and (c) getting all to participate. Our third category involves getting parents that often are underrepresented to come, for example, women with birth fears, foreign-born parents and fathers. It mainly concerns preparation and organisation of a group beforehand and not an actual group session. It also has no subcategories and was excluded from further analysis. All subcategories of the first two main categories are presented in Table 1.

The qualitative analysis also resulted in three main categories of *rewards* of working with parental groups: (a) well-functioning groups, (b) personal benefit and (c) development of the individual in the group. The first category concerns aspects connected to creating conditions for a well-functioning group. The second category focuses on oneself as the leader of a parental group, and personal gains of being in that role. The third category involves seeing development in individual group members (as opposed to a group development as in the first category). The third category concerns aspects which are not directly linked to groups, and it was therefore excluded from further analysis. The subcategories of main categories one and two are presented in Table 2.

Based on the summative content analysis, comparing levels of experience for those represented in a subcategory (those who expressed the difficulty or reward) with those who were not showed a pattern where significant differences were found within each main category (see Table 3). For the main category ‘managing group processes’, those expressing difficulties in managing emotions and creating security within the group were more experienced, whereas for the other three subcategories there were no differences. For the main category ‘uncomfortable in the role’, those expressing difficulties in finding the right level of content and managing professional shortcomings were less experienced.

The leaders finding creating conditions for cohesion and lasting friendship rewarding were more experienced (main category 'well-functioning groups'). For the main category ‘personal benefit’, those expressing it rewarding to get to know the parents and getting confirmation from them were less experienced.

Finally, an investigation of possible patterns of difficulties and rewards as a function of experience was conducted. All subcategories for difficulties and rewards separately were ordered based on the median difference of experience between the leaders expressing the category and those who did not. This ordering showed which difficulties the leaders were preoccupied with – from least to most experienced (Figure 1) and the same for rewards (Figure 2). The rank correlations for difficulties and experience as a whole, r(355) = .29, BCa 95% CI [.20, .39], and for rewards, r(355) = .27, BCa 95% CI [.16, .36], were both positive and significant. There was also a significant and positive rank correlation between rewards and difficulties, r(355) = .21, BCa 95% CI [.10, .32], showing those ranking high on rewards also ranked high on difficulties.

## Discussion

The results showed that different levels of experience can be connected to different perceptions of difficulties and rewards when working as a leader for parental education groups. The more experienced leaders were more preoccupied with aspects of getting the group to work as well as possible. This involves managing emotions, that is, managing the influence of individual parents in terms of expressions of anxiety or sadness, but also intense questioning of the group or leader, or extreme opinions perceived as inappropriate in the group. The more experienced leaders expressed creating a safe, open and tolerant climate as most difficult. Less experienced leaders were more preoccupied by issues connected to themselves, their professional competence and content to be used in the group.

What the leaders saw as rewarding when working with parental education groups also varied depending on their experience. Experience per se is not a guarantee of skill acquisition; however, a learning process involves experience of the practice to progress in skill acquisition according to Dreyfus and Dreyfus (2009). Issues involving the parent group itself and how it works were predominant among the more experienced leaders, whereas issues involving their own role occupied the less experienced leaders. The most experienced leaders found gratification in contributing to and fostering cohesion, as well as creating conditions for lasting friendship and a continuing network between parents even after the group was over. The least experienced leaders focused on getting to know the parents and receiving positive confirmations from them that the meetings work and that the parents are satisfied.

These differences could be understood in terms of skill acquisition. The skill and competence as a leader and teacher of a parental education group in terms of the Dreyfus model (Dreyfus and Dreyfus, 1986, 2004, and 2009) for more experienced and less experienced leaders are different. The least experienced leaders may be more dependent and reliant on a set of rules and instructions created by someone else (such as an instructional manual) in both planning and delivery of parental education groups. The situational perception of a leader with little experience is limited, and complexity in the situation is more likely to be seen as hindrance more than a challenge. The more experienced leader may still rely on some form of instructional manual, but has an ability to use it differently, depending on the context. It may be understood as an ability to recognise the relevance of specific cues in the interaction with parents and to see what is important in the situation.

Previous research has shown a lack of academic or scientific knowledge as well as training connected to a leader role in this context (Barlow et al., 2009; Lefevre et al., 2015; Sarkadi, 2009). In a normal nursing practice outside parental education groups, there is a mix of scientific knowledge and rules of thumb acquired with experience (Dreyfus and Dreyfus, 2009). The skills necessary to be a leader of parental education groups do not currently include much of the scientific knowledge from nursing school (Forslund Frykedal and Rosander, 2015). This makes the specific situation of being a group leader dependent on rule of thumb to a larger degree. Acquisition of relevant skills does not include many opportunities to learn by observation as group leadership is often done in isolation from other nurses or midwives, so leaders are in many cases left to trial and error or an instructional manual (Dreyfus and Dreyfus, 1986).

The rank order for the subcategories of difficulties can be understood in terms of each subcategory as a prerequisite for the next one. This can be understood in terms of the Dreyfus model where skills of one stage need to be acquired before progressing to the next stage in the learning process (Dreyfus and Dreyfus, 1980). Being able to handle one’s own role as a leader is necessary to be able to work successfully with a group, and creating involvement is necessary to be able to create security. Managing emotions and creating security require the leader to be sensitive to the group and work with it based on the specific situation. This requires a higher level of skill and competence. The results show a pattern of increase with each subcategory for the experienced leaders and a decrease for the less experienced. *Creating security* is almost exclusively a challenge for the experienced leaders. For the less experienced leaders, this may not even be something they consider as they are preoccupied by lower levels of difficulties, such as, what to talk about in the group and how to be a leader.

For the subcategories of the rewards, a similar pattern can be discerned. The results indicate a hierarchy of rewards depending on experience, each connected to specific skills and stages in the Dreyfus model (Dreyfus and Dreyfus, 1986). A focus on one’s own role and a basic interaction with parents, getting to know them and getting positive feedback on one’s role as leader are prerequisites for the continuing work with the group. For the least experienced leaders, this is rewarding enough. For more experienced leaders, this activity is not seen as a reward. They find rewards from working with the group creating conditions for cohesion and lasting friendship. This requires the leader to take in and work more with the specific conditions of the current group and, at least to some degree, set some long-term goals in relation to actions taken. Even a novice or an advanced beginner could possibly attain the first two subcategories following a manual. However, creating conditions for cohesion and lasting friendship require a higher skill level in line with theory (Dreyfus and Dreyfus, 1986, 2004, and 2009).

The less experienced leaders’ emphasis on one’s own role is what would be expected based on the Dreyfus model. All nurses and midwives in this study have some experience of working with parental groups but, as they often do it in solitude without supervision (Forslund Frykedal et al., 2019), it is easy to get stuck at a level of competence and skill. A reasonable assumption is that we find a majority of the less experienced leaders in Dreyfus Stage 1 or 2, that is, the novice and advanced beginner (Dreyfus and Dreyfus, 2004) in terms of their pedagogical and group psychological skills, and a majority of the more experienced leaders in Dreyfus Stage 3 and above. Of course, some of the more experienced leaders, no doubt, will still be at the first stages of skill acquisition, and some of the less experienced leaders will have progressed towards higher stages of competence. However, for the less experienced this is more likely based on individual aptitude or persistence and personal interest in these issues. For the more experienced stuck at a lower level of skill and competence in leading a group, a reasonable explanation might be lack of specific training and supervision (cf. Forslund Frykedal et al., 2019). Supervision can improve awareness and sensitivity in the professional role (Severinsson et al., 2010). Lefevre et al. (2015) reported that leaders of parental groups see a need for supervision as well as training in group leadership. Without supervision and competence training, it is more difficult to progress unless individual nurses or midwives take a special interest in the problem and make sure they themselves are prepared for the challenges involved in working with and leading a group to provide high quality health promotion.

A limitation of this study is that the data are from nurses and midwives all working in a large city in Sweden. Even though the Swedish way of continuing with parental education groups after the perinatal period is somewhat different when compared to the practice in other countries, the focus of this study was not on a specific method of delivering parental education, but on more general aspects connected to being a leader of these groups. The estimate of experience is a rough estimate, but the closest we can come to a real measurement of experience from our data. It assumes that the number of groups a leader starts each year and the number of times they meet a group are roughly the same throughout their professional career. This may vary; however, we believe our estimate comes closer than just relying on general work experience.

Based on the results of the current study, a controlled intervention using a tailored training programme dependent on the experience of the participants could test the effectiveness and consequences for parents as well as the confidence of the leaders.

### Relevance to clinical practice

This study highlights issues important for nurses and midwives in the role as leaders with different experiences which can be a starting point to identify important pedagogical and group psychology skills needed to work with parental education groups. Tailoring specific training for nurses and providing (collegial) supervision when working with groups would help less experienced leaders to progress towards higher levels of skills in leading parental education groups. This is likely to increase the number of parents benefiting from being part of such groups. In the long run, having this type of training systematically in nurse education as well as in continuing education for nurses would be something for which to strive.

The results are also relevant to and can be transferred to any clinical practice in which a nurse works in a leader role with groups of patients, for example, working with lifestyle changes in diabetes groups or hypertension groups. Taking the pedagogical and group psychology aspects into account have the potential to not only be a way to save resources, but also to provide care and patient education of high quality.

## Conclusions

Overall, our results show a focus on one’s own role and how one can learn or benefit from working with a parent group for less experienced leaders, and with experience, comes a greater focus on the group itself and how to make it a well-functioning group. Not being able to take the current group and the specific context into account when working as a leader for a group reduces the possibility of achieving a well-functioning group and a parent-centred learning environment. Consequently, the goals of parental education, that is, to strengthen parents’ competence as parents and to provide for a continuing network among parents will be harder to reach. The challenge is to find ways for less experienced leaders to progress towards higher levels of skill in leading parental groups. To feel secure in the leader role when working with parental education groups – being able to create balance and security in groups, providing conditions for cohesive groups in which one as a leader has sufficient skills and capacity to manage interference and emotions arising – increases not only the possibility for successful parental education groups, but also results in a better work environment for midwives and nurses.
